# Prenatal care with blind pregnant women: validation of an instrument for nurses on knowledge, attitude and practice

**DOI:** 10.17533/udea.iee.v43n3e11

**Published:** 2025-11-06

**Authors:** Francisco Jardsom Moura Luzia, Monaliza Ribeiro Mariano Grimaldi, Kariane Gomes Cezário Roscoche, Josemara Barbosa Carneiro, Antonia Ellen Jardani de Souza Medeiros, Liliana Andreia Neves da Mota, Cristina Maria Correia Barroso Pinto, Paula Marciana Pinheiro de Oliveira

**Affiliations:** 1 Nurse, Ph.D. student. Email: jardsommouraenf@aluno.unilab.edu.br. Corresponding author. https://orcid.org/0000-0002-8386-6103 Universidade da Integração Internacional da Lusofonia Afro-Brasileira Brazil jardsommouraenf@aluno.unilab.edu.br; 2 Nurse, Ph.D. Professor of the Graduate Program in Nursing. Email monalizamariano@unilab.edu.br. https://orcid.org/0000-0002-8718-4783 Universidade da Integração Internacional da Lusofonia Afro-Brasileira Brazil monalizamariano@unilab.edu.br; 3 Nurse, Ph.D. Professor of the Nursing Department. Email: karianeroscoche@ufpr.br. https://orcid.org/0000-0002-2097-2478 Universidade Federal do Paraná Brazil karianeroscoche@ufpr.br; 4 Nurse, Ph.D. student. Email: josemarabarbosac@gmail.com. https://orcid.org/0000-0003-4650-9809 Universidade da Integração Internacional da Lusofonia Afro-Brasileira Brazil josemarabarbosac@gmail.com; 5 Nurse, master's student. Email: jardanimedeiros@hotmail.com. https://orcid.org/0000-0003-1974-2090 Universidade da Integração Internacional da Lusofonia Afro-Brasileira Brazil jardanimedeiros@hotmail.com; 6 Nurse, Ph.D. RISE-Health Teacher. Email: liliana.mota@essnortecvp.pt. https://orcid.org/0000-0003-3357-7984 Northern School of Health of the Portuguese Red Cross Portugal liliana.mota@essnortecvp.pt; 7 Nurse, Ph.D. Professor of the Nursing Department. Email: cmpinto@esenf.pt. https://orcid.org/0000-0002-6077-4150 Universidade do Porto Portugal cmpinto@esenf.pt; 8 Nurse, Ph.D. Professor of the Graduate Program in Nursing. Email: paulapinheiro@unilab.edu.br. https://orcid.org/0000-0001-9091-0478 Universidade da Integração Internacional da Lusofonia Afro-Brasileira Brazil paulapinheiro@unilab.edu.br; 9 University of International Integration of Afro-Brazilian Lusophony, Brazil. Universidade da Integração Internacional da Lusofonia Afro-Brasileira University of International Integration of Afro-Brazilian Lusophony Brazil; 10 Federal University of Paraná, Brazil. Universidade Federal do Paraná Federal University of Paraná Brazil; 11 Northern School of Health of the Portuguese Red Cross, Portugal. Northern School of Health of the Portuguese Red Cross Northern School of Health of the Portuguese Red Cross Portugal; 12 Porto School of Nursing, Portugal. Universidade do Porto Porto School of Nursing Portugal

**Keywords:** health knowledge, attitudes, and practice, prenatal care, nursing care, primary health care, people with visual impairments., conocimientos, actitudes y práctica en salud, atención prenatal, atención de enfermería, atención primaria de salud, personas con daño visual., conhecimentos, atitudes e prática em saúde, cuidado pré-natal, cuidados de enfermagem, atenção primária à saúde, pessoas com deficiência visual.

## Abstract

**Objective.:**

To verify evidence of validity of the instrument “Knowledge, Attitude, and Practice of Nurses in Prenatal Care for Visually Impaired Pregnant Women”.

**Methods.:**

Methodological study to assess content validity with the participation of 22 expert nurses (11 from the area of Sexual and Reproductive Health and 11 from the area of People with Disabilities). The instrument was adapted into a Google Forms questionnaire and assessed Objectivity, Clarity, and Relevance. The data were analyzed using the Content Validity Coefficient and Content Validity Ratio, in addition to calculating Cronbach's Alpha for internal consistency. Ten generalist nurses from Primary Care participated in the semantic validation, evaluating comprehension, relevance, and possible adjustments to the instrument, with calculation of the Semantic Concordance Index.

**Results.:**

The instrument obtained an overall Content Validity Coefficient above 0.90 for objectivity, clarity, and relevance, with internal consistency (Cronbach's Alpha = 0.89). The experts' suggestions improved the wording and structure. In semantic validation, the Semantic Concordance Index was 0.97, reinforcing clarity and applicability.

**Conclusion.:**

The instrument showed evidence of content validity, being objective, clear, and relevant for assessing the Knowledge, Attitude, and Practice of nurses in the prenatal care of pregnant women with visual impairment.

## Resumen

**Objetivo.:**

Evaluar la validez del instrumento “Conocimientos, Actitudes y Prácticas de Enfermeras en la Atención Prenatal de Embarazadas con Discapacidad Visual”.

**Métodos.:**

Estudio metodológico que analizó la validez de contenido con la participación de 22 enfermeras especialistas, 11 del área de Salud Sexual y Reproductiva y 11 del área de Discapacidad. El instrumento se aplicó en Google Forms y se evaluaron la objetividad, claridad y relevancia de cada una de sus preguntas. Se utilizaron el Coeficiente y la Razón de Validez de Contenido, además del Alfa de Cronbach para evaluación de la consistencia interna. También se realizó validación semántica con la participación de diez enfermeras generalistas del área de Atención Primaria, considerando comprensión, pertinencia y ajustes, a través del Índice de Concordancia Semántica.

**Resultados.:**

El instrumento alcanzó un Coeficiente de Validez de Contenido superior a 0.90 en los tres criterios y un Alfa de Cronbach de 0.89, indicando consistencia interna adecuada. Las sugerencias de los expertos contribuyeron a mejorar la redacción y la estructura. En la validación semántica, el Índice de Concordancia Semántica fue de 0.97, confirmando claridad y aplicabilidad.

**Conclusión.:**

El instrumento mostró evidencia sólida de validez de contenido, siendo objetivo, claro y relevante para evaluar conocimientos, actitudes y prácticas de enfermeras en la atención prenatal de embarazadas con discapacidad visual.

## Resumo

**Objetivo.:**

Verificar evidências de validade do instrumento de “Conhecimento, Atitude e Prática de Enfermeiros no pré-natal de gestantes com deficiência visual”.

**Métodos.:**

Estudo metodológico para avaliar a validade de conteúdo com a participação de 22 enfermeiros especialistas (11 da área de Saúde Sexual e Reprodutiva e 11 da área de Pessoas com Deficiência). O instrumento foi adaptado em formulário no *Google Forms* e avaliou Objetividade, Clareza e Relevância. Os dados foram analisados por meio do Coeficiente de Validade de Conteúdo e Razão de Validade de Conteúdo, além do cálculo do Alpha de Cronbach para consistência interna. Dez enfermeiros generalistas da Atenção Primária participaram da validação semântica, avaliando compreensão, relevância e possíveis ajustes no instrumento, com cálculo do Índice de Concordância Semântica.

**Resultados.:**

Instrumento obteve Coeficiente de Validade de Conteúdo global acima de 0.90 para objetividade, clareza e relevância, com consistência interna (Alpha de Cronbach = 0.89). As sugestões dos especialistas aprimoraram redação e estrutura. Na validação semântica, o Índice de Concordância Semântica foi 0.97, reforçando clareza e aplicabilidade.

**Conclusão.:**

O instrumento apresentou evidências de validade quanto ao conteúdo, sendo objetivo, claro e relevante para avaliar o Conhecimento, Atitude e Prática de enfermeiros no pré-natal de gestantes com deficiência visual.

## Introduction

Sexual and reproductive health care for women with disabilities has often been surrounded by stigmas that directly impact the care provided to this population. However, the equitable process that guides the Unified Health System (SUS) requires professionals to be prepared to serve all individuals, regardless of color, race, ethnicity, disability, or vulnerability, necessitating methods that promote comprehensive care.^1^ The lives of women with visual impairments are still hampered by attitudinal barriers on the part of professionals and the community, which reinforce the precept that people are asexual.^2^ Furthermore, ableism persists, permeating discourse that characterizes them as incapable of fully developing the role of motherhood, or even of gestation, due to a limited view of their disability, without considering desires and plans these women.^3^

Besides issues related to attitude, the technical and care practices of health professionals, including nurses, face limitations related to their educational path. The lack of classes and content related to the care of Persons with Disabilities (PwD) constitutes a significant gap and can directly impact the quality of services provided to this population.^4^ In this context, the development of Knowledge, Attitude, and Practice (KAP) assessment instruments emerges as a relevant tool, as it allows for the assessment of gaps in knowledge, behaviors, or attitudes that can become barriers to care. Furthermore, it can identify irregularities in the care provided, enabling interventions to be established that facilitate practices free from prejudice, discrimination, and ableism.^5^

To ensure an accessible and effective healthcare system, the development of instruments that identify the main gaps in core aspects of prenatal care (Knowledge, Attitude, and Practice) and that present evidence of validity could contribute to the advancement of nursing practices for people with disabilities. Furthermore, it will help to advance policies aimed at guaranteeing the sexual and reproductive rights of the public and enable a situational assessment to promote continuing education methods for professionals. Furthermore, it is noteworthy that no other KAP instrument related to Persons with Disabilities has been identified in the literature, nor with specificity for prenatal consultations, thus presenting an innovative character. Therefore, this study aims to verify evidence of the validity of the Knowledge, Attitude, and Practice of Nurses instrument in the prenatal care of pregnant women with visual impairment.

## Methods

This methodological study developed and evaluated the validity of a KAP instrument for assessing nurses' prenatal consultations with women with visual impairments. The study was conducted in three stages: 1) Selection, content delimitation, and construction of the KAP instrument; 2) Assessment of content validity evidence; and 3) Assessment of semantic validity evidence. The first stage consisted of delimiting the content used in the construction of the KAP instrument and was based on the Nonverbal Communication Model for Nursing Consultations with blind Patients,^6^ the Verbal Communication Model with the Blind: Development and Validation in Consultations with the blind patient,^7^ and the Healthcare Guide for Women with Disabilities and Reduced Mobility^8^ for inquiries directed at consultations with people with disabilities, and the Ministry of Health's Low-Risk Prenatal Care Handbook^9^ for inquiries related to prenatal consultations. 

In the second stage, judges from the Prenatal and Persons with Disabilities areas assessed content validity evidence. In the third stage, Primary Care nurses conducted semantic evaluation, the target audience for the instrument.

Data collection for the study was conducted online via email and Google Forms between July 2023 and March 2024. The study population consisted of experts in the areas of Persons with Disabilities and Sexual and Reproductive Health, as well as nurses working in Primary Care.

Regarding the experts, the inclusion criteria for defining participants were defined based on attributes that encompassed the experts' academic and professional characteristics. Recruitment initially began with research groups in the areas of health promotion for people with and without vulnerabilities and Sexual and Reproductive Health promotion. Subsequently, the CVs of the previously selected experts were analyzed on the Lattes Platform, through the Portal of the National Council for Scientific and Technological Development (CNPq), and a search was carried out using the terms "Person with Disabilities", "Person with Visual Impairment", "Sexual and Reproductive Health" and "Prenatal" to complement the necessary number. In total, 46 experts were invited via email and instant messaging through the social network WhatsApp, which was part of the researchers' contact network: 16 from the area of ​​People with Disabilities and 30 from the area of ​​Sexual and Reproductive Health; however, 24 experts were discontinued for not confirming participation. The final panel consisted of 22 judges, considering the stratification of 11 from the area of ​​Sexual and Reproductive Health and 11 from the area of ​​People with Disabilities.^10^ A deadline of 20 days was established for the experts to respond to the validation instrument. Those who completed the response within this period were included in the sample, discontinuing those who did not return or who left the validation instrument incomplete.

For generalist PHC nurses, we chose to include those with at least one year of experience conducting prenatal consultations. Convenience sampling was conducted through contact via instant messaging on the social network WhatsApp, using the referral network technique. Based on the literature, the number of participants in this stage should be between 10 and 30. For this study, the recommended reference framework was followed, with assessments performed by 10 members of the target audience.^11^

To characterize expert nurses, the following sociodemographic variables were selected: gender, age, current occupation, time since graduation, highest degree, care experience in the areas covered by the instrument, and research conducted in the area in the last five years. In turn, to characterize nurses, the following sociodemographic variables were selected: gender, age, marital status, time since graduation, and time in practice, provision of prenatal consultations for women with visual impairments, and presence of women with visual impairments in their area of​expertise.

Regarding the variables involved in content validation, all questions were assessed for objectivity, clarity, and relevance. For semantic validation, the following variables were defined: perception of the instrument, understanding of the questions, response options, importance of the questions, changes and additions to the instrument, and questions not answered. To assess content validity evidence, an invitation letter was sent to the experts, presenting the study's information, objectives, and methodology. After confirmation of participation, the data collection instrument, adapted as a Google Forms® form, was made available. It was structured in three parts: 1^st^. Informed Consent Form; 2^nd^. Questionnaire for the judges' sociodemographic and professional characteristics; 3^rd^. An adapted content validation instrument assessed objectivity (direct, concise, and aligned with the proposal), clarity (understandable and unambiguous wording), and relevance (relevant to the topic and contributing to practice) of each proposed item, using a Likert scale with "Yes," "No," and "Partially" options, as well as a field for additional suggestions from the judges.^12^

To assess the semantic validity evidence, a form was sent to the nurses via Google Forms®, also structured in three parts: 1^st^. Informed Consent Form; 2^nd^. Questionnaire for the sociodemographic and professional characterization of the nurses; 3^rd^. Version of the instrument revised after content validation, in addition to a semantic validation instrument adapted from the DISABKIDS semantic validation instrument in Portuguese.^13^ For content validation, the Content Validity Coefficient (CVC) was used, calculating the individual coefficient (CVCi) and the total CVC (CVCt). Only questions with inter-expert agreement equal to or greater than 0.80 were considered valid.^14^ The Content Validity Ratio (CVR) was also calculated, assessing inter-rater agreement on the instrument's items using a three-point scale: 3 - Essential to the instrument; 2 - Useful, but not essential to the instrument; and 1 - Not necessary. The critical CVR value considered for the study was 0.418, defined based on the number of experts who participated in the study and a significance level of 0.05.^15^

For semantic validation, the proportion of inter-expert agreement was analyzed using the Semantic Agreement Index (SIC), which assesses relevant characteristics of the instrument.^16^ Items were considered semantically validated when they reached an agreement greater than 70% (0.70). An analysis of the construct's internal consistency was also performed using Cronbach's alpha calculations with a 95% confidence interval. For this study, a Cronbach's alpha coefficient equal to or greater than 0.70 was considered acceptable.^17^

All study participants signed the Informed Consent Form and received a copy. The project was submitted to and approved by the Research Ethics Committee (REC), under report number 6.168.208 e CAAE n° 705324423.3.0000.5576.

## Results

Once the components for the instrument were selected, adaptations were made to meet the specific needs of prenatal consultations for women with visual impairments, resulting in the first version of the survey. This initial version of the instrument included 40 questions: 13 in the knowledge domain, 13 in the attitude domain and 14 in the practice domain, with two subjective questions. The instrument then underwent content validation.

The content validation stage involved 22 judges, 11 of whom were experts in the area of ​​Persons with Disabilities and 11 in the area of ​​Sexual and Reproductive Health. Of these, 19 (86.36%) were women, with a higher concentration in the 30-40 age group (59.09%) and predominantly in teaching (72.73%). It was also observed that the majority of participants had between 11 and 15 years of training (50%). All sample members held master's degrees, of which 17 (77.27%) held PhD. The same number reported having healthcare experience in the areas covered by the KAP instrument. All had conducted research in the field within the last five years.

Regarding the individual CVC of all the evaluators, 21 items achieved complete agreement among the evaluators across the three criteria assessed (CVCi=1.00). Furthermore, 38 items presented results within the acceptability parameters adopted for the study (>0.80), and only two presented lower results, as shown in Table 1. 

**Table 1:** Content Validity Indices by dimension and items of the KAP Instrument of the 22 expert judges

Content Validity Indices				
		Objectivity			Clarity			Relevance
Dimension/ Number	Content	CVC	RVC	CVC	RVC	CVC	RVC
Knowledge							
1	Types of Visual Impairment	1.00	1	1.00	1	0.95	0.82
2	Visual impairment as a risk factor	1.00	1	0.98	0.91	1.00	1
3	Accessibility	0.93	0.73	0.84	*0.45*	1.00	1
4	Segregation	0.98	0.91	0.95	0.91	0.98	0.91
5.	Presence of a companion.	1.00	1	1.00	1	1.00	1
6	Ableism	1.00	1	0.93	0.73	1.00	1
7	Sexual and reproductive rights.	0.95	0.82	0.95	0.82	0.98	0.91
8	Changes in the consultation environment.	1.00	1	0.95	0.82	1.00	1
9	Chair position during the consultation.	1.00	1	1.00	1	1.00	1
10	Intimate or Close Personal Distance.	1.00	1	0.93	0.73	1.00	1
11	Observation of facial and body expressions.	0.93	0.82	0.82	*0.36*	0.89	0.73
12	Presentation of palpation materials.	1.00	1	0.95	0.91	1.00	1
13	Restrictions on motherhood.	0.80	*0.18*	*0.73*	*0.09*	0.95	0.82
Attitude							
14	The nurse should stand and the patient should sit during the consultation.	0.84	*0.45*	*0.77*	*0.36*	1.00	1
15	Direct questions to the companion.	1.00	1	1.00	1	1.00	1
16	Greet the pregnant woman at the door and escort her to the room.	1.00	1	1.00	1	1.00	1
17	Introduce yourself to the pregnant woman without introducing the other professionals.	1.00	1	1.00	1	1.00	1
18	Provide audio description before the consultation.	0.98	0.91	0.98	0.91	0.89	0.73
19	Raise your voice.	1.00	1	1.00	1	1.00	1
20	Describe the room.	1.00	1	1.00	1	1.00	1
21	Maintain eye level with the visually impaired pregnant woman.	1.00	1	1.00	1	1.00	1
22	Refer the pregnant woman to high-risk prenatal care.	1.00	1	1.00	1	0.91	0.82
23	The presence of a companion is essential.	1.00	1	1.00	1	0.95	0.82
24	Provide guidance on motherhood.	1.00	1	1.00	1	1.00	1
25	Indicate physical touch.	1.00	1	1.00	1	1.00	1
26	Silence after each question.	1.00	1	1.00	1	0.95	0.91
27	In professional practice, referrals to high-risk prenatal care are common.	1.00	1	1.00	1	1.00	1
Practice							
28	Maintains effective verbal communication.	1.00	1	1.00	1	1.00	1
29	Accessible health education activities (Subjective).	0.98	0.91	0.98	0.91	1.00	1
30a	Collects health, family, and gynecological history.	1.00	1	1.00	1	1.00	1
30b	Explains the procedure that will be performed before the examination.	1.00	1	1.00	1	1.00	1
30c	Explains test results and serology in an understandable manner.	1.00	1	1.00	1	1.00	1
30d	Explains folic acid and ferrous sulfate supplementation in an understandable manner.	1.00	1	1.00	1	1.00	1
30e	Explains the scheduling of necessary vaccinations in an understandable manner.	1.00	1	1.00	1	1.00	1
30f.	Explains vaginal delivery in an understandable manner.	1.00	1	1.00	1	1.00	1
30g	Explains cesarean delivery in an understandable manner.	1.00	1	0.98	0.91	1.00	1
30h	Explains signs of labor onset in an understandable manner.	1.00	1	1.00	1	1.00	1
30i	Explains the stages of labor in an understandable manner.	1.00	1	1.00	1	1.00	1
30j	Explains risk signals in a comprehensible way.	1.00	1	1.00	1	1.00	1
31	Strategies used to maintain effective communication (Subjective)	1.00	1	0.98	0.91	0.95	0.82
CVC Global		0.98		0.99		0.98	

CVC: Content Validity Coefficient, CVR: Content Validity Ratio

Considering the CVR scores, only question 13 scored below the critical value recommended for the study (0.18) for objectivity, and questions 11, 13, and 14 scored below the critical value recommended for clarity (0.36; 0.09; 0.36). All questions scored higher for relevance. Regarding the overall CVR of the evaluations conducted by all experts, the instrument presented agreement scores above 0.90 for all items, exceeding the recommended level for the study (0.80). Cronbach's alpha test was used to assess the instrument's internal consistency, which demonstrated high reliability across all items (α=0.89).

After evaluating each item, space was provided for suggestions. This identified weightings that were considered in the composition and structure of the evaluated questions. All recommendations were evaluated and, when deemed relevant to improving the instrument, were accepted. The main suggestions were to change the wording of some questions to improve understanding, changes in the dimension to which the question belonged and the insertion of practical examples in the questions that addressed definitions as shown in Table 2.

**Table 2:** Suggestions from the 22 content judges and results of the researchers' analysis

Question	Suggestions made by experts	Accepted
03	Introduce elements of the nurse's daily life to better understand what "urban planning, architecture, communication" means.	Yes
04	Include the term/word/synonym for segregation in parentheses.	Yes
06	Include the term/word/synonym for ableism in parentheses.	Yes
08	Include changes in positioning so the nurse understands the type of change being referred to.	Yes
09	Although it does require prior knowledge, I believe that the way of acting and behaving toward pregnant women with visual impairments is more appropriate to the attitude domain.	No
10	If the value of distance is part of a standard, I suggest a multiple-choice question to assess whether the professional would know how to assess the position between himself and the patient.	No
13	I found the question confusing. I suggest rewording it and checking if any words or punctuation need to be changed to make it clearer.	Yes
14	Rewrite the question to be more objective.	Yes
28	The professional's concept of effective communication can influence the answer. I suggest conceptualizing effective communication applied to PwD.	No

The experts' suggestions resulted in the second version of the KAP assessment instrument for nurses in prenatal consultations for visually impaired pregnant women, which remained with 40 items and underwent semantic validation. The semantic validation stage involved 10 PHC nurses, eight (80%) of whom were women, aged between 20 and 30 years (50%), and single (60%). Furthermore, the results were equal regarding time since graduation (one to five years) and five to ten years (50%), and a predominance of one to three years in the field (50%). Furthermore, all participants in the sample had already provided prenatal consultations in their care practice. Two (20%) had already provided prenatal consultations for visually impaired pregnant women, and six (60%) were unable to say whether or not there were women with visual impairments in their area of ​​practice. The results obtained from the Semantic Concordance Index (SIC) showed an overall concordance of 97% and all items presented scores above 90%, as shown in Table 3.

**Table 3:** Semantic Concordance Index (SCI) of the KAP Instrument of the 10 Primary Health Care nurses

Items	Response	**Agreement (*n*)**	**Disagreement (*n*)**	SCI (%)
What did you think of the instrument?	Very good/good	10	0	100
Did you understand the questions?	Easy to understand	9	1	90
Did you have difficulty with the answer options?	No	9	1	90
Are the questions important for assessing KAP in prenatal care for visually impaired pregnant women?	They are very important	10	0	100
Would you change anything in the instrument?	No	10	0	100
Would you add anything to the questionnaire?	No	10	0	100
Would you not like to answer any questions?	No	10	0	100
General Semantic Agreement Index				97

## Discussion

The KAP assessment instrument for nurses providing prenatal care to women with visual impairments showed consistent validity evidence in the evaluation of experts in the field of sexual and reproductive health and people with disabilities, with an overall CVC above 0.98 in objectivity, clarity, and relevance, indicating statistically significant agreement on the comprehensiveness of the content selected for the questionnaire. For the individual CVC of the questions, there was significant agreement for most items. Only one item presented a poor score in objectivity and two in clarity, but it was decided to modify and maintain the instrument due to the favorable results in relevance. Similar results were observed in the evaluation of the validity evidence of the KAP instrument on COVID-19 preventive measures for prison staff, which presented a CVC above 0.96 in all items evaluated, demonstrating consistency in the validation process.^18^

Regarding the experts, despite the prevalence of teaching as an occupation (72.73%), a significant portion reported having healthcare experience in the field (77.27%). The use of expert selection criteria that valued not only academic training but also practical experience contributed to the improvement of the instrument, enabling closer ties to the healthcare context inherent to the construct's objective.^19^ It is noteworthy that the presence of experts with extensive healthcare experience in the instrument's areas of interest, combined with their academic training, allows for accurate assessment, ensuring the improvement of the developed construct and ensuring the credibility of the version that will later be administered to the target audience.^20^

Furthermore, regarding the CVR evaluation, the experts considered most of the questions essential for the instrument, considering the critical values ​​established for the study. However, three questions presented inferior results: one for objectivity and clarity, and two for clarity only. Because they received positive evaluations for relevance, the items remained in the instrument after adjustments for semantic validation. Similar results and procedures were observed in the development and validation of content for a website for patients with coronary artery disease, which presented CVR results above the cut-off mean for most of the website's content items. However, those that did not reach these results were evaluated based on the experts' suggestions and remained for subsequent lay evaluation.^21^

Therefore, to assess the instrument's internal consistency based on the experts' assessment, Cronbach's alpha was calculated, which demonstrated results that ensure high consistency in the combination of questions. In this regard, a study that evaluated the psychometric properties of the dropout factors scale in undergraduate nursing courses showed similar internal consistency in the sum of all items, and it was considered capable of analyzing the situations that influence dropout.^22^ Before the instrument was subjected to semantic validation, all opinions expressed by experts aimed at improving the instrument's questions were considered, and those deemed valid by the researchers were accepted. After the modifications that resulted in the second version of the KAP instrument, semantic validation was performed, which consisted of an evaluation by a portion of the instrument's target audience. Regarding the sociodemographic characterization variables, the sociodemographic profile of PHC nurses in the state of Paraiba, northeastern Brazil, showed that of the 462 professionals, just over 93% were female, in contrast to the predominant age group of 36 to 40 years. Another factor that differed from that found in this study was marital status, with a prevalence of married individuals.^23^

In the context of knowledge in the field, it is essential that nurses identify all the social, economic, and health conditions of their assigned population during the territorialization process, so that actions can be planned and agreed upon that ensure the universality advocated by the principles of the Unified Health System (SUS).^24^ Regarding the semantic evaluation of the questions, the results show that for the target audience, the instrument is good/very good, and the questions are easy to understand, as are the answer options. Furthermore, everyone considered the questions important and assessed KAP in prenatal care for women with visual impairments. When asked if they would change or add anything, or if there were any questions they did not want answered, the unanimous answer was "no." Furthermore, no suggestions were made.

The results showed an overall SCII of 97%, which represents excellent semantic agreement. A similar outcome was observed in a study that produced and validated a podcast to promote mental health among primary care users, which obtained an SCI of 95%.^25^ Therefore, this study hopes to contribute to the improvement of nursing practices aimed at prenatal care for women with visual impairments. This instrument, with evidence of consistent validity, will allow for a situational analysis of care and subsequent implementation of interventions that will ensure comprehensive actions and reduce barriers that directly interfere with motherhood on an equal footing with other women without disabilities.

As a limitation, following the methodological framework, the instrument still needs to undergo further validation processes with a more representative sample of the target audience, which is the proposed follow-up to the study.

The conclusion of this study is that the instrument presents consistent validity evidence regarding content, being considered objective, clear, and relevant for assessing nurses' KAP in prenatal care for women with visual impairments. Furthermore, the questions were considered essential for the instrument, presenting a CVR above the cutoff mean in most questions and considered semantically clear and easy to understand by a portion of the target audience, demonstrating the effectiveness of the methodological procedures adopted for the study.

## References

[1] Seidu AA, Malau-Aduli BS, McBain-Rigg K, Malau-Aduli AE, Emeto TI (2024). Sexual lives and reproductive health outcomes among persons with disabilities: a mixed-methods study in two districts of Ghana. Reproductive Health.

[2] Machado CV (2024). Democracy, citizenship and health in Brazil: challenges to strengthening the Unified Health System (SUS). Ciência e saúde coletiva.

[3] Qi W, Li H, Lian Q, Zuo X, Yu C, Lou C, Tu X (2023). Knowledge level and access barriers related to sexual and reproductive health information among youth with disabilities in China: a cross-sectional study. Reproductive Health.

[4] Mheta D, Sibiya MN, Nkosi PB (2023). Experiences of Women with Disabilities in Accessing Maternal Healthcare Services: A South African Case Study. International Journal of Environmental Research and Public Health.

[5] Cameron V, Roth J (2024). Disability Through a Nursing Professional Development Lens. Journal for Nurses in Professional Development.

[6] Huang A, Hong W, Zhao B, Lin J, Xi R, Wang Y (2022). Knowledge, attitudes and practices concerning catheter‐associated urinary tract infection amongst healthcare workers: a mixed methods systematic review. Nursing Open.

[7] Rebouças CBA, Pagliuca LM, Sawada NO, Almeida PC (2012). Validation of a non-verbal communication protocol for nursing consultations with blind people. Revista Rene.

[8] Costa KN (2009). Modelo de comunicação verbal com o cego: desenvolvimento e validação em consulta de enfermagem [Dissertation]: Repositório Institucional da UFC.

[9] Brasil. Ministério da Saúde, Secretaria de Atenção à Saúde (2019). Guia de Atenção à Saúde das Mulheres com Deficiência e Mobilidade Reduzida.

[10] Brasil. Ministério da Saúde, Secretaria de Atenção à Saúde, Departamento de Atenção Básica (2012). Atenção ao pré-natal de baixo risco.

[11] Pasquali L (2013). Psicometria: Teoria e aplicações.

[12] Chaves TD, Guedes TG, Perrelli JG, Albuquerque NL, Mangueira SD, Linhares FM (2023). COVID-19 nas prisões: validação de um inquérito de conhecimento, atitude e prática. Acta Paulista de Enfermagem.

[13] Carona C, Crespo C, Silva N, Lopes AF, Canavarro MC, Bullinger M (2013). Examining a developmental approach to health-related quality of life assessment: Psychometric analysis of DISABKIDS generic module in a Portuguese sample. Vulnerable Children and Youth Studies.

[14] Coelho FU, Reigota SM, Cavalcanti FM, Regagnin DA, Murakami BM, Santos VB (2024). Bladder ultrasound: evidence of content validity of a checklist for training nurses. Revista Brasileira de Enfermagem.

[15] Wilson FR, Pan W, Schumsky DA (2012). Recalculation of the Critical Values for Lawshe’s Content Validity Ratio. Measurement and Evaluation in Counseling and Development.

[16] Scaratti M, Johann GR, Argenta C, Zanatta EA (2023). Content and semantics validation of an application for adolescents with diabetes mellitus. Acta Paulista de Enfermagem.

[17] Giacomini I, Martins MR, Matijasevich A, Cardoso MA (2023). Internal consistency of the Strengths and Difficulties Questionnaire in Amazonian children. Revista de Saúde Publica.

[18] Chaves TD, Guedes TG, Perrelli JG, Albuquerque NL, Mangueira SD, Linhares FM (2024). COVID-19 in prisons: validity of a knowledge, attitude and practice survey. Acta Paulista de Enfermagem.

[19] Melo ESJ, Silva MJN, Silva APN, Braga HFGM, Oliveira BSB, Monteiro FPM, Barbosa LP (2024). Criteria for selecting experts in the evaluation of educational technologies in Nursing: an integrative review. Revista Rene.

[20] Gomes DS, Souza PA, Assis GM, Paula DG (2024). Validation of a mobile application for adults with neurological lower urinary tract dysfunction. Revista Latino Americana de Enfermagem.

[21] Arroio LF, Lopes JD, Barros AL, Lima EA, Lopes CT, Santos VB (2023). Desenvolvimento e validação de conteúdo de um website para pacientes com doença arterial coronariana. Revista Brasileira de Enfermagem.

[22] Mattos LM, Barlem ELD, Barlem JGT (2024). Propriedades psicométricas da escala de fatores de evasão nos cursos de graduação em Enfermagem. Educação por escrito.

[23] Alvarenga JD, Sousa MF (2023). Processo de trabalho de enfermagem na Atenção Primária à Saúde no estado da Paraíba - Brasil: perfil profissional e práticas de cuidados na dimensão assistencial. Saúde em Debate.

[24] Duarte BAR, Leite TCP, Felix TR, Santana MAO, Giuliani CD (2023). Home visits in rural areas: demands in primary care. Rural Remote Health.

[25] Girardi KH, Zanatta L, Zocche DAA, Vendruscolo C, Hirdes A (2024). Production and validity of a podcast to promote mental health among primary care users. Revista de Enfermagem da UFSM.

